# Toward an all-in-one recombinant adeno-associated virus vector for functionally ablating the prion gene using CRISPR-Cas technology

**DOI:** 10.1371/journal.pone.0336578

**Published:** 2025-11-07

**Authors:** Claire Verkuyl, Ari Belotserkovsky, Thomas Zerbes, Declan Williams, Medha R. Krishnan, Sabrina Zhu, Sophie Grunnesjӧ, Shehab Eid, Cunjie Zhang, Wenda Zhao, Leo Xu, Eleanore Lin, Teaghan O’Shea, Benjamin Draper, Andreas Jungman, Patrick Most, Gerold Schmitt-Ulms

**Affiliations:** 1 Tanz Centre for Research in Neurodegenerative Diseases, University of Toronto, Toronto, Ontario, Canada; 2 Department of Laboratory Medicine & Pathobiology, University of Toronto, Toronto, Ontario, Canada; 3 Megadalton Solutions, Inc., and Department of Chemistry, Indiana University, Bloomington, Indiana, United States of America; 4 German Centre for Cardiovascular Research (DZHK), Heidelberg, Germany; 5 Department of Internal Medicine III Cardiology, University of Heidelberg, Heidelberg, Germany; Fudan University, CHINA

## Abstract

Any strategy that can selectively and persistently lower the brain levels of the cellular prion protein (PrP^C^) is expected to extend survival in prion diseases. Recent advances in the virus-mediated delivery of gene therapies prompted us to explore if a recombinant adeno-associated virus (rAAV) vector delivering a CRISPR-Cas-based gene editor can be devised that induces a functional knockout of the prion gene. Whereas the eventual objective is to assess the therapeutic potency of an optimized vector in prion-infected mice, in this proof-of-concept study, we evaluated tools and methods that are suited to achieve this goal. The result of these efforts is a first-generation all-in-one rAAV vector that codes for a prion gene-specific guide RNA and a small Cas9 endonuclease, whose expression is controlled by a truncated neural cell adhesion molecule 1 (NCAM1) promoter that is active in PrP^C^ expressing cells. We also constructed a second rAAV vector coding for a prion gene-specific ‘traffic light reporter’ (TLR). The TLR can be used to monitor prion gene-editing efficacy by coding for red and green fluorescent proteins separated by a segment of the prion gene that is targeted by the gene editor. For the purification of AAVs, we adopted a robust and scalable rAAV vector assembly pipeline and undertook proof-of-concept prion gene editing experiments in human cells and mice, which to date yielded prion gene editing rates of approximately 20% and 5%, respectively. Finally, we compared brain distributions of rAAV vectors following intrathalamic versus retro-orbital injection, and selected the 9P31 capsid for future studies based on a 7.5-fold higher heterologous gene expression level as compared to the PHP.eB capsid.

## Introduction

Prion diseases are rapidly progressive fatal dementias that afflict a subset of vertebrates and are the cause of death in approximately one in five thousand humans [1]. In prion diseases, the cellular prion protein (PrP^C^) aggregates into β-sheet-rich stacked assemblies [2–4], named PrP Scrapie (PrP^Sc^) after the first known prion disease in sheep [5]. What causes a small number of individuals—yet not others—to develop these diseases is not understood, except when the disease is passed on through ingestion of PrP^Sc^-tainted foods or medical treatments [6]. Moreover, in approximately 15% of human cases [1], the disease is inherited in the form of coding variants in the prion gene (*PRNP*) that render the mutated PrP^C^ more prone to form PrP^Sc^.

The evolutionary origins of the prion gene [7] and the distribution and interactions of PrP^C^ point toward a function in the control of the polysialylation of NCAM1 [8]. Consistent with this notion, ablation of NCAM1 polysialylation or deficiency of the prion gene cause highly similar and mild phenotypes in mice [9]. The mild consequences of *Prnp*-ablation extends to cattle that was rendered *Prnp*-deficient [10], a goat that was observed to be naturally devoid of a functional *Prnp* gene [11], and a few humans who were discovered to have one of their *PRNP* alleles functionally ablated [1].

Taken together, these characteristics of the prion gene and the central role assigned to its gene product in the etiology of prion diseases suggest that an effective and safe treatment may aim to lower PrP^C^ expression. Recent work, first with antisense oligonucleotides (ASOs), and subsequently with divalent siRNAs targeting *Prnp* transcripts in prion-infected mice robustly validated this therapeutic objective [12,13].

For these treatments to be effective in the clinic, ASOs or divalent siRNAs need to penetrate deeply into the human brain. Clinical trials based on ASOs or divalent siRNAs targeting prion protein RNAs have been initiated to assess this characteristic in humans. Prior reports on other ASOs indicate this to be a formidable hurdle [14,15]. A shared additional challenge of ASOs or siRNAs represents their inherently transient nature. With these agents, peak-trough effects are observed that require repeat intrathecal injections for prolonged suppression [16].

In parallel to work with ASOs and divalent siRNAs, impressive prion disease survival extension has been reported with recombinant adeno-associated virus (AAV)-delivered gene therapies that either silence the transcription of *Prnp* alleles in mouse brains with customized *Prnp*-specific zinc finger repressors (ZFRs) [17,18] or through methylation of *Prnp* promoter sequences by an epigenetic editor [19]. A shared challenge of both approaches represents their inherently transient nature. Although relative to ASOs a considerably slower decline in effectiveness is expected based on data which indicate that multi-year expression of rAAV vector delivered payloads can be achieved [20], their method of PrP ablation is transient. If repeat injections of rAAV vectors were required, robust immunoreactivity would have to be expected that may render consecutive treatments ineffective or unsafe [21]. This caveat was most recently addressed with the help of rAAV-delivered base editors, which were directed to the *Prnp* ORF to generate permanent edits by converting specific cytosine and adenine codons into nonsense codons. Using their most potent base editing system, the authors documented an impressive 63% reduction in average PrP levels in the mouse brain [22].

For now, rAAV vector-based treatments of neurodegenerative diseases that engulf much of the human brain are mainly hindered by limitations in the widespread transduction of brain cells.

In recent times, methodologies for optimizing the tropism of rAAV vector capsids have expanded [23,24], leading to several groups reporting advanced capsids that can be administered systemically and mediate dramatically improved access to the brain in old world monkeys (see, for instance [18,25–28]). Parallel to these developments, smaller CRISPR-Cas endonucleases have been identified that can be accommodated in all-in-one rAAV vectors [29], including Cas9 endonucleases from *Staphylococcus aureus* [30], *Neisseria meningitides* [31,32], and *Staphylococcus lugdunensis* [33]. Finally, two Phase I clinical studies have made headlines by demonstrating that the lipid nano particle-based delivery of Cas9 endonucleases and guide RNAs to liver cells can achieve remarkable reductions of specific gene products. The first study of this kind targeted the gene that codes for the blood circulating protein transthyretin (TTR), which can cause transthyretin amyloidosis (ATTR). Specifically, it documented that harnessing the host cell-encoded, error-prone non-homologous end-joining (NHEJ) program can lower transthyretin blood levels by >90% in clinical trial subjects [34]. A conceptually similar approach was reported just as we were beginning to assemble this manuscript. It lowered expression of the kallikrein B1 gene, a validated therapeutic target for the treatment of hereditary angioedema, with CRISPR-Cas9-based gene editing eliminating 95% of plasma kallikrein levels in patients who received the highest dose [35].

Inspired by these advances, here we report on the early phase development of a gene therapy based on all-in-one rAAV vectors whose payload we designed to functionally ablate the prion gene in brain cells using an NHEJ-reliant gene editing approach. We engineered separately murine and human gene editing vectors and adapted a rAAV vector purification pipeline for this purpose. We then show how the *in vivo* transduction efficacy or our all-in-one rAAV vectors can be determined in cells and mouse brains by using a bespoke traffic light reporter (TLR) or next-generation amplicon sequencing of the edited prion gene. Finally, we compared different routes of administration and rAAV capsids which had been reported to have a more potent neuronal tropism in C57Bl/6 mice.

## Materials and methods

### Antibodies

#### Primary antibodies.

For immunoblotting we used the recombinant humanized fragment antigen-binding region D18 against PrP (epitope: non-linear, centered on residues 133–157 in mouse PrP) [36], generously provided by the laboratory of Dr. Emil F. Pai (University of Toronto, Toronto, ON, Canada), at a dilution of 1:5,000, and the mouse monoclonal IgG1 anti-hemagglutinin (HA) tag antibody (clone 2-2.2.14, catalog number 26183, Thermo Fisher Scientific, Waltham, MA, USA) against the HA tag that was C-terminally fused to the SluCas9-HF endonuclease, at a dilution of 1:2,000. To standardize total loaded protein, a beta-actin horseradish peroxidase (HRP)-conjugated monoclonal antibody was used at a dilution of 1:30,000 (catalog number MA5–15730-HRP, Thermo Fisher Scientific).

For IHC, the primary antibodies included rabbit anti-Prion protein PrP (clone EP1802Y) (catalog number ab52604, Abcam, Cambridge, UK) at a 1:600 dilution, rat anti-GFAP (catalog number 13–0300, Invitrogen, Waltham, MA, USA) at 1:1000, and mouse anti-NeuN (catalog number MAB377, Millipore Canada Ltd, Etobicoke, ON, Canada) at 1:100.

#### Secondary antibodies*.*

Goat anti-human IgG kappa light chain recombinant secondary antibody conjugated to HRP, at 1:2,000 dilution (catalog number A56864 Thermo Fisher Scientific). Goat anti-mouse IgG polyclonal HRP antibody, at 1:5,000 dilution (catalog number 31430, Thermo Fisher Scientific). Goat anti-mouse IgG heavy and light chain cross-adsorbed HRP conjugated antibody, at 1:5,000 dilution (catalog number 31432, Thermo Fisher Scientific).

### Cloning

#### NCAM1-SluCas9-HF-gRNA construct assembly.

To replace the cytomegalovirus (CMV) promoter with the truncated NCAM1 promoter, the plasmid coding for SluCas9-HF (catalog number 163796, Addgene, Watertown, MA, USA) was double digested with restriction endonucleases XbaI and NcoI (catalog numbers 10128088 and 10161944, New England Biolabs Ltd., Whitby, ON, Canada). The products of the restriction digest were subsequently loaded onto a 1% agarose gel, followed by gel extraction and purification of the plasmid backbone without promoter (catalog numbers T1020L and T1030S, New England Biolabs). In parallel, the 611 bp segment of the NCAM1 promoter that is proximal to the transcription start site (nucleotide range −611 to −1) was amplified through polymerase chain reaction using primers that contained 5’ XbaI and 3’ NcoI restriction sites (see **Table 1** for primers). Next, the amplicons were ligated to the linearized SluCas9-HF plasmid by T4 ligase (catalog number EL0014, Thermo Fisher Scientific), the ligation product transformed into NEB stable bacteria (catalog number C3040H, New England Biolabs), colonies amplified in lysogeny broth medium supplemented with ampicillin and sequence verified.

**Table 1:** Primers for NCAM1-SluCas9-HF-MM1, TLR, and CBh-EGFP scAAV assembly.

Primer Name	Sequence (5’→3’)
Forward NCAM1 (Xba1 insertion site)	ctagtctagagaatcgaaatggagggattt
Reverse NCAM1 (Nco1 insertion site)	catgccatggtaatctgctggctggga
Forward mCherry	ccagccagcagattaccatggatggtgagcaagggcgag
Reverse mCherry	tctgccctcaccggatcccttgtacagctcgtccatgcc
Forward mGreenLantern	tggtgacgtgagcaagggcgaggag
Reverse mGreenLantern	atcagcgagctctaggttacttgtacagctcgtccatgtca
MM1 gRNA sequence	agggtggaacaccggtggaa
CP gRNA sequence	tttgtgactatgtggactgatc
Forward EGFP	caggttggaccggctagcaccggtgccaccatgaagctgggccg
Reverse EGFP	gtaatccagaggttgattaggaagttcatccttctacttgtacagctcgtccatgccg

The gRNAs specific for the human *PRNP* or mouse *Prnp* open reading frames (ORFs) were inserted 3’ of the U6 promoter and immediately upstream of the coding sequence for the CRISPR scaffold RNA present in the SluCas9-HF plasmid using the Q5 Site-Directed Mutagenesis Kit (catalog number E0554S, New England Biolabs), and the accuracy of the insertion was confirmed by DNA sequencing.

#### Traffic light reporter construct assembly.

SluCas9-HF was excised from the NCAM1-SluCas9-HF plasmid by restriction endonucleases NcoI and EcoRI (catalog numbers R0101L and R3101, New England Biolabs). Following the restriction, the backbone of the plasmid was extracted from an agarose gel and purified. In parallel, genes coding for monomer Cherry (mCherry) or monomer GreenLantern (mGreenLantern) were PCR amplified from deposited plasmids (catalog numbers 20596 and 161912, Addgene) (see **Table 1** for primers), and synthetic phosphorylated single-stranded DNA oligos were obtained that coded for the *PRNP*- or *Prnp*-specific protospacers, a SluCas9-HF compatible protospacer adjacent motif (PAM)—conforming to its NNGG requirement—, and a termination codon. Next, a three fragment assembly inserted the synthetic construct and the mGreenLantern sequence into the linearized plasmid coding for the truncated NCAM1 promoter with the help of the NEB HiFi DNA Assembly Cloning Kit (catalog number E5520, New England Biolabs). Subsequently, a restriction digest with endonuclease Nco1 opened the construct, and the mCherry ORF with Nco1-digested overhangs was ligated 5’ of the synthetic segment, followed by transformation of NEB stable bacterial cultures.

To insert the chicken-β-actin (CBh) promoter into the TLR plasmid, double restriction digests of the TLR plasmid and a plasmid comprising the CBh promoter (catalog number 194245, Addgene) were undertaken with endonucleases XbaI and NcoI. Finally, the agarose gel extracted bands, coding for the backbone of the TLR plasmid, and the excised CB promoter were ligated, transformed into NEB stable bacterial cultures, and their sequence verified by DNA sequencing.

#### CBh EGFP scAAV construct assembly.

The CBh promoter and self-complimentary ITR AAV backbone plasmid was deposited to Addgene by the Michael J Fox Foundation MJFF (catalog number 194245, Addgene). The EGFP sequence was PCR amplified from its donor plasmid (catalog number 22875, Addgene) using primers detailed in **Table 1** in Q5 Hot Start High Fidelity 2X Master Mix (catalog number M0494, New England Biolabs) according to manufacturer instructions. The backbone vector was digested with AgeI-HF and StuI (catalog numbers R3552 and R0187, New England Biolabs) simultaneously according to manufacturer instructions. This removed the ‘null’ sequence and linearized the plasmid. Then, the backbone was purified on a 0.9% agarose gel. The EGFP gene was inserted into the linearized plasmid using NEB HiFi Assembly 2X Master Mix (catalog number E2621, New England Biolabs) relying on overlap regions inserted at the ends of each PCR primer. The final plasmid was transformed into NEB Stable Competent E. coli (catalog number C3040H, New England Biolabs) and sequence verified.

### Cell culture

#### NMuMG, U2OS, and HeK293 cell culture and maintenance.

Cells were proliferated in growth medium consisting of Dulbecco’s Modified Eagle Medium (DMEM) (catalog number 11995073, Thermo Fisher Scientific) supplemented with 10% fetal bovine serum (catalog number 12484028, Thermo Fisher Scientific), 1% Glutamax (catalog number 35050061, Thermo Fisher Scientific), and 1% penicillin-streptomycin (catalog number 15140122, Thermo Fisher Scientific). To expand the cultures upon reaching 80% confluency, cells were rinsed with phosphate buffered saline, detached with trypsin and passaged 1:10.

### rAAV purification

The production and purification of rAAV vectors was based on a previously reported method [37]. Briefly, HEK293T were grown in a HYPERFlask with 1,720 cm^2^ surface area and 560 mL growth medium (CLS10031−4EA, MilliporeSigma). At 70−80% confluency, the cells were triply transfected with the rAAV transfer plasmid, the AAV packaging plasmid coding for Rep and Cap genes, and the helper plasmid coding for adenovirus genes E2A, E4 and VA. Five days later, 3 mL Triton-X 100 (catalog number X100, Sigma-Aldrich, Oakville, ON, Canada), 250 µl RNAse A (catalog number EN0531, Thermo Fisher Scientific, Mississauga, ON, Canada), 56 µl Pluronic F-68 (catalog number 24040−032, Thermo Fisher Scientific), and 56 µl Turbonuclease (catalog number T4330: Sigma-Aldrich) were added to the cell culture medium in the HYPERFlask. This was followed by agitation of the flask at 150 rpm and 37°C for 1 hour. The cell lysates were then removed from the HYPERFlask, before rinsing the HYPERFlask with 140 mL of Dulbecco’s phosphate buffered saline (PBS) (catalog number 14190−144, Thermo Fisher Scientific). The lysate and PBS wash were then pooled and spun down at 4,000 g for 30 minutes to create a pellet of cell debris. The centrifugation supernatant was filtered through a 0.45 µm PES Autofil bottle top vacuum filter assembly of 1L (catalog number 1143-RLS, Foxx Life Sciences, Londonderry, NH, USA).

The filtrate was subsequently loaded onto a POROS GoPure AAVX pre-packed column (catalog number A36652, Thermo Fisher Scientific) at a rate of 0.2-0.3 mL/min over 44 hours using an ӒKTA fast protein liquid chromatography (FPLC) system (GE Healthcare, Chicago, IL, USA). Before eluting the rAAV, the affinity matrix was subjected to four washes at a rate of 0.5 mL/min as follows: 15 mL of 1 × Tris-buffered saline (TBS) (0.05 M Tris/HCl, pH 7.6, 0.15 M NaCl), 15 mL of 2 × TBS, 20 mL of 1x TBS with 20% ethanol (v/v), and 15 mL of 1 × TBS. 12 mL of elution buffer consisting of 0.2 M glycine and 0.01% Pluronic F-68, pH 2-2.5, was used to elute the rAAV vectors across three 4 mL fractions, each collected in a tube containing 420 µL of neutralization buffer (1 M Tris/HCl, pH 8, 0.1% Pluronic F-68). Prior to use, each buffer was filtered through a 0.22 µM PES membrane with a Stericup Quick Release-GP filtration system (catalog number S2GPU11RE, MilliporeSigma Canada Ltd., Oakville, ON, Canada).

The three elution fractions were then pooled and diluted with final formulation buffer (0.001% Pluronic F-68, 35 mM NaCl, and 1 × PBS) to a total volume of 50 mL. The rAAV vectors were then filtered through a 0.22 µM PES Millex-GP Syringe Filter Unit (catalog number SLGPR33RS, MilliporeSigma Canada Ltd.) before concentration with an Amicon stirred cell ultrafiltration unit (catalog number UFSC05001, MilliporeSigma Canada Ltd) with a 100 kDa Ultrafiltration Disc (catalog number PLHK04310, MilliporeSigma Canada Ltd). The Amicon stirred cell ultrafiltration unit was attached to a nitrogen tank and placed on a magnetic stir plate set to a low to medium speed. The volume of the AAV vector concentrate was allowed to reach 5 mL before the Amicon stirred cell ultrafiltration unit was refilled with final formulation buffer to 50 mL. This step was repeated three times in total, and on the last concentration the contrate was reduced to 1 mL. The rAAV vectors were then removed from the Amicon stirred cell ultrafiltration unit with a pipette for storage in 20–50 µL aliquots at −80°C. Any glass or plasticware in contact with rAAV vectors was coated with 0.005% Pluronic F-68, 35 mM NaCl, 1 × PBS before use.

### rAAV titration

To determine the titer of our rAAV preparations we followed a protocol previously outlined by Challis et al. based on qPCR quantitation of rAAV genomes [38]. First, we prepared DNA standards as follows: We linearized 20 µg of the respective transfer plasmid with the restriction enzyme ScaI (catalog number R3122S, New England Biolabs) and ran the product on a 0.9% agarose gel to confirm linearization. A DNA cleanup kit (catalog number T1030S, New England Biolabs) was used to remove impurities from the digested DNA, before eight serial dilutions of this standard stock were completed in triplicate using UltraPure water (catalog number 10977−015, Invitrogen).

When preparing our rAAVs for titration, we began with a DNAse I digestion (catalog number EN0521, Thermo Fisher Scientific) at 37°C for 1 hour in digestion buffer (2 mM CaCl_2_, 10 mM Tris-HCl, and 10 mM MgCl_2_). 5U of DNAse I were used per 2 µl of virus in a 100 µl reaction. DNAse I was then deactivated by adding 5 µl EDTA (0.5 M, pH 8.0) (catalog number 15575−038, Invitrogen) to each tube before incubating for 10 minutes at 70°C. The product was then digested with Proteinase K (catalog number PRK403.100, BioShop Canada Inc., Burlington, ON, Canada) to release viral genomes from their capsids. This digestion was accomplished by adding 120 µl of buffer composed of 1 M NaCl and 1% (wt/vol) N-lauroylsarcosine sodium salt and 12 µg of Proteinase K to the DNAse digestion product. This was incubated at 50°C for at least 2 hours. Proteinase K was then deactivated for 10 minutes at 95°C. These final rAAV samples were then diluted 1:300 in UltraPure water. qPCR reactions were conducted in triplicate with each containing the following: 12.5 µl SYBR Green Master Mix (catalog number A46012, Thermo Fisher Scientific), 9.5 µl UltraPure water, 0.5 µl each of the forward and reverse primer at 2.5 µM initial concentration, and 2 µl of the diluted sample. Respective primers can be found in **Table 2**. A LightCycler 480 Instrument II (Roche Life Science Solutions, Basel, Switzerland) was used to run samples first for 10 minutes at 95°C, then for 40 cycles of 15 seconds at 95°C and 60 seconds at 60°C.

**Table 2:** rAAV titration primers.

Primer Name	Sequence (5’ →3’)
Forward CBh EGFP scAAV	cctattgacgtcaatgacgg
Reverse CBh EGFP scAAV	gatgtactgccaagtaggaaag
Forward Traffic Light Reporter	gctgaccgcccaacgac
Reverse Traffic Light Reporter	gtactgccaagtaggaaagtccc
Forward NCAM1-SluCas9-HF-MM1	ctgggactggacatcggaatc
Reverse NCAM1-SluCas9-HF-MM1	gcgtaggggtttgtagactgagg

### Animals

Animal studies were necessary to assess the delivery and expression of rAAV vector in intact brains. To this end, C57BL/6 mice (Jackson Laboratory, Bar Harbor, ME, USA) were housed with an artificial 12 hr day/night cycle in cages with no more than five mice. Drinking water for the mice was available *ad lib*, food was a protein chow, daily health checks were performed, and cage changes occurred once a week. Procedures and animal care were performed in accordance with the Canadian Council on Animal Care, reviewed and authorized by the University Health Network Animal Care Committee and approved under Animal Use Protocol 6840. All staff handling the animals or undertaking surgical procedures underwent specific training for the respective tasks to ensure the humane handling of the animals. The study was designed to assess the expression of the rAAV-delivered payload, using three mice per cohort. Initial cohorts comprised mice that were transduced by stereotaxic injection with AAV9-encapsulated rAAV vectors coding for EGFP, the mouse prion gene-specific traffic light reporter TLR-MM1, the therapeutic SluCas9-HF-MM1 payload, or both the TLR-MM1 and SluCas9-HF-MM1. Later experiments were based on rAAV vectors whose capsids exhibit improved BBB penetrance, including PHP.eB and 9P31, and were delivered through retro-orbital intravenous injection. All rAAV vectors were administered to mice that were deeply sedated with 5% isoflurane inhalation through a nose cone. During prolonged sedation, the isoflurane level was reduced to 2%. To assess the desired level of deep sedation, the animals were evaluated for pain response using the toe pinch reflex (pedal reflex). During surgery, respiration was observed to ensure a regular respiratory pattern. All mice were sacrificed 21 days after stereotaxic or retro-orbital injection. This timeline was selected to allow the transduced rAAV vector payloads to express fully. Together, the study made use of approximately 50 mice, of which 10% died during deep anesthesia as an inadvertent outcome of surgical procedures. None of the mice that survived the stereotaxic or retro-orbital injection died subsequently before the end of the 21 day observation window due to late effects of the surgery or illness.

### Stereotaxic injections

Stereotaxic surgery was conducted with a Stoelting quintessential stereotaxic injector (QSI) (catalog number 53311, Stoelting Co., Wood Dale, IL, United States) and a customized 10 µL cemented needle syringe (Model 701, Point Style: 4, 33 gauge, needle length: 25 mm, 45° angle, Hamilton, Reno, NV, USA). Once the mice were heavily anesthetized by isoflurane inhalation, the heads of the mice were shaved, and ophthalmic ointment applied to the eyes before positioning them in the QSI. Meloxicam (5 mg/kg) was administered subcutaneously, then the skin on the head was wiped with betadine and 70% ethanol. An incision was then made along the midline of the scalp and the connective tissue above the bone was gently scraped away using the blunt edge of a scalpel handle. Next, the QSI was adjusted so that a customized 10 µL Hamilton syringe aligned with bregma, followed by moving it 1 mm to the right and 1.7 mm posteriorly before a mark was made on the skull. A 23 gauge needle was used to make a hole in the skull at this position before the Hamilton syringe was lowered to 3.5 mm below the skull. 2x 10^11^ viral genomes in a maximum volume of 2.5 µL were injected into the ventral lateral nuclei of the thalamus at a rate of 100 nL per second before the Hamilton syringe was slowly withdrawn. For ICV injections the same procedure was followed, but the needle was positioned at 0.5 mm to the right and 1 mm posterior to bregma before lowering 3 mm below the skull. The Hamilton syringe remained in the brain for 3 minutes before it was slowly withdrawn. The incision site was then cleaned and sutured, and 1 mL of saline administered subcutaneously, before recovery on a heating pad.

### Retro-orbital injections

In preparation of retro-orbital injections, mice were anesthetized with 5% isoflurane using a nose cone. Next, one eyeball of the mice was slightly displaced from the eye socket through the application of gentle pressure to the skin, dorsal and ventral to the eye by spreading the eyelids with the index finger and thumb without applying excessive pressure to the ventral cervical vessels. Following local anesthesia through a drop of ophthalmic solution (0.5% proparacaine hydrochloride), the injection needle was carefully introduced at a 45^0^ angle into the medial canthus, then advanced until its tip reached the base of the eye. During the slow injection of 100 µL of rAAV vectors, the needle was gradually pulled back, then completely removed. Following the injection, a second droplet of ophthalmic solution was administered to avoid infections. After the injection, mice were returned to their cages for recovery. Following the procedure, the mice were monitored for bulging of the eye, cornea injury, or inflammation.

### *In vitro* transfection or transduction of cells and fluorescence detection

Transfection was performed using Lipofectamine 3000 (catalog number L3000001, Thermo Fisher Scientific) and 500 ng of DNA in OptiMEM (catalog number 31985062, Thermo Fisher Scientific) according to the manufacturer’s instructions. 1x10^5^ cells were seeded on a 24 well glass imaging plate (catalog number P24-1.5P, Cellvis, Mountain View, CA, USA) and proliferated to ~70–80% confluence prior to transfection. Transduction of cells was undertaken by adding rAAV vectors at an MOI of 1x10^6^ vg copies/cell to the cell culture medium. Fluorescent *in vitro* transfection images of cells were obtained with a Zeiss Axio Observer microscope. Cells were imaged at magnifications of 10x (0.65 µm/pixel) and 40x (0.16 µm/pixel), detecting wavelengths of 592–667 nm and 500–550 nm for green and red fluorescence, respectively.

### Cryo-sectioning and *in vivo* fluorescence detection in brain slices

Following deep anesthetization with inhaled isoflurane, mice underwent two-minute transcardiac perfusion with PBS prior to five-minute perfusion with 10% formalin (catalog number HT501128, Sigma-Aldrich). Brains were then dissected and placed in 10% formalin for four hours. Subsequently, brains were briefly rinsed with PBS before their transfer to 30% sucrose and incubation for 36 hours. Tissue-Tek optimum cutting temperature (O.C.T.) compound (catalog number 4583, Sakura Finetek, Torrance, CA, USA) was used for freezing, which was accomplished through partial submersion of brains in 2-methylbutane that had been cooled with liquid nitrogen.

Next, brains were coronally sliced with a cryostat (HM525 NX, Thermo Fisher Scientific), whose temperature was held at −25°C, beginning 4 mm posterior to bregma and travelling anteriorly. 20 µM or 50 µM slices were mounted onto Superfrost Plus Slides (catalog number 48311–703, VWR International, Radnor, PA, USA). Two brain slices from the same region and animal were mounted on the same slide and secured using mounting medium with DAPI (catalog number ab104139, Abcam, Cambridge, UK) under the cover slip. Fluorescence was visualized on a Zeiss Axioscan microscope at 10x or 20x magnification (0.65 or 0.5 µm/pixel, respectively). DAPI signals, green and red fluorescence were detected at wavelengths of 435–485 nm, 500–550 nm, and 592–667 nm, respectively.

### Immunohistochemistry

For immunofluorescence staining, tissue sections were first de-paraffinized and subjected to heat-induced epitope retrieval using citrate buffer (pH 6.0) in a pressure cooker. To block non-specific antibody binding, the sections were incubated with Protein Block, Serum Free (catalog number X0909, Agilent, Santa Clara, CA, USA). Following a wash with 1 × Tris-buffered saline containing 0.1% Tween-20 (TBST), the sections were incubated for one hour at room temperature with Mouse on Mouse (M.O.M.) Blocking Reagent (catalog number MKB-2213–1, Vector Laboratories, Newark, CA, USA). Primary antibody incubation was performed overnight at 4°C using a cocktail prepared in Dako Antibody Diluent (catalog number S3022, Agilent).

The following day, sections were washed with TBST and incubated for one hour at room temperature with a secondary antibody cocktail, also diluted 1:200 in Antibody Diluent. This cocktail consisted of goat anti-rabbit Alexa Fluor 555 (catalog number A32732, Invitrogen), goat anti-rat Alexa Fluor 647 (catalog number A48265, Invitrogen), and goat anti-mouse Alexa Fluor 488 (catalog number A11029, Invitrogen). After another TBST wash, sections were counterstained with DAPI (catalog number D9542, Sigma-Aldrich) for five minutes. To minimize tissue auto fluorescence, the sections were treated with a saturated solution of Sudan Black B in 70% ethanol (catalog number 199664, Sigma-Aldrich) for 20 minutes at room temperature. Finally, coverslips were mounted using Vectashield Vibrance Antifade Mounting Medium (catalog number H-1700, Vector Laboratories).

### Western blotting

To prepare samples for western blotting, brains were perfused, or cells were washed with ice-cold PBS, followed by lysis with buffer containing 0.5% NP40, 0.5% DOC, 150 mM NaCl, 150 mM Tris/HCl, pH 8.3, plus protease and phosphatase inhibitors. Following lysis, the samples were spun down to pellet cellular debris and supernatants were collected. Protein concentrations were then determined using the bicinchoninic acid (BCA) colorimetric method (catalog number 23225, Thermo Fisher Scientific). Next, samples were adjusted to have equal protein before loading onto Bis-Tris denaturing sodium dodecyl sulfate polyacrylamide gel electrophoresis (SDS-PAGE) gels (catalog number NW04125BOX, Thermo Fisher Scientific) and subsequent transfer to polyvinylidene fluoride (PVDF) membranes (catalog number IPVH00010, Millipore Canada Ltd, Etobicoke, ON, Canada). Following the protein transfer, membranes were blocked with 5% skim milk in Tris-buffered saline supplemented with Tween 20 (TBS-T) before they were probed with primary antibodies overnight at 4°C. Subsequently, PVDF membranes were washed with TBS-T three times for ten minutes each, followed by incubation with the respective secondary antibody for one hour at room temperature. Finally, three more ten-minute TBS-T washes were performed before western blots were reacted with Western Lightning ECL Pro chemiluminescence reagents (catalog numbers 0RT2505 and 0RT2405, PerkinElmer, Waltham, MA, USA) and signals were detected on X-ray films.

### Next-generation sequencing (NGS) of prion gene amplicons

In biological triplicates, U2OS and HEK293T cells were seeded on a 6-well plate (83.3920) and allowed to reach ~80% confluency 1 day before transfection. Cells were transfected with the NCAM1-SluCas9-HF plasmid using lipofectamine 3000 as per the manufacturer’s instructions, followed by incubation at 37C and 5% CO2. In parallel, three mice were injected with the rAAV-NCAM1-SluCas9-HF vector. Genomic DNA was extracted from cells 3 days post-transfection and mouse brains 3 weeks post-injection using the NEB Monarch Genomic DNA Purification Kit (T3010S), following the manufacturer’s instructions. Purified genomic DNA was then delivered to the Donnelly Sequencing Center for downstream processing, beginning with amplicon generation using *PRNP*- or *Prnp*-specific primers (**Table 3**). 150-paired end amplicon sequencing was carried out by the Illumina MiSeq instrument, with 100,000 reads allocated per sample. Sequences collected were analyzed using CRISPResso to determine the frequency and type of insertion-deletion mutations generated after gene editing [39].

**Table 3:** Next-generation sequencing primers.

	Forward	Reverse
*PRNP*	TCGTCGGCAGCGTCAGATGTGTATAAGAGACAGgctggatgctggttctctttgtggc	GTCTCGTGGGCTCGGAGATGTGTATAAGAGACAGcaccaccgccctgaggtgggtag
*Prnp*	TCGTCGGCAGCGTCAGATGTGTATAAGAGACAGggccctctttgtgactatgtggactg	GTCTCGTGGGCTCGGAGATGTGTATAAGAGACAGggctgcccccaggtgc

### Charge detection mass spectrometry (CD-MS)

Prior to CD-MS analysis, frozen rAAV vector preparations were thawed at 4 ⁰C, before being buffer exchanged into 200 mM ammonium acetate (catalog number AM9070G, Thermo Fisher Scientific), 0.01% F68 (catalog number 24040032, Thermo Fisher Scientific) in UltraPure DNase/RNase-Free Distilled Water (catalog number 10977015, Thermo Fisher Scientific) using Micro Bio-Spin P-6 gel columns (catalog number 7326225, Bio-Rad). All samples were ionized and introduced to the CD-MS instrument by TriVersa NanoMate chip-based electrospray ionization technology (Advion Interchim Scientific, Ithaca, NY, USA). CD-MS measurements used an electrostatic linear ion trap instrument (Megadalton Solutions, Inc.). The instrument was configured to transmit a broad mass range at an ion energy of 130 eV/z and a trapping time of 104.6 ms, yielding uncertainty of ~0.8 e (elementary charges).

### Statistical analyses

Quantification of all Western blot data was based on densitometry analysis (ImageJ, National Institutes of Health and the Laboratory for Optical and Computational Instrumentation, University of Wisconsin, WI, USA). The Microsoft Excel Analysis ToolPack and GraphPad Prism were used to compute two-sample, equal variance t-tests. For tallying signals in fluorescent images, the number of cells fluorescing red, green, or both red and green were counted manually in randomly selected fields of view. All error bars represent one standard deviation. Values of p < 0.05 were considered statistically significant. Results of significance tests are indicated in graphs as follows: ns = p > 0.05, p * = p < 0.05, ** = p < 0.01, *** = p < 0.001.

## Results

### Assembly of all-in-one rAAV vector for the functional ablation of the prion gene

**Fig 1:**
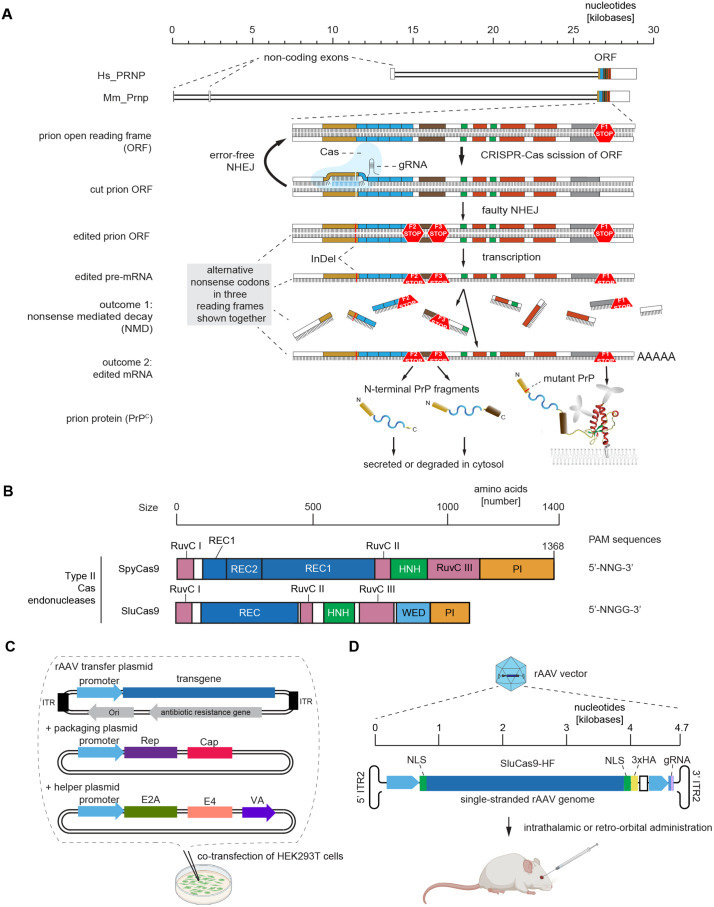
Assembly of all-in-one rAAV vector for the functional ablation of the prion gene. **(A)** Cartoon depicting the prion gene with enlarged view of the 5’ end of its ORF and the possible consequences of NHEJ-induced frameshifts leading to premature termination codons (PTCs). The latter may trigger the destruction of mRNAs harboring indels by nonsense-mediated decay (NMD) or their translation into short N-terminal PrP fragments that terminate prematurely. **(B)** Comparison of the relative sizes, domain organizations, and protospacer adjacent motifs of SpyCas9 and SluCas9-HF. **(C)** Components of the rAAV vector production pipeline used in this study. **(D)** Organization of the rAAV vector for the functional ablation of prion genes, showing to scale the origins of its components including the size of its promoters, the organization of the SluCas9-HF expression cassette, and the relative orientation and sizes of elements that drive the expression of the prion gene directed gRNA. Abbreviations: NLS and 3xHA, designate the positions of nuclear localization sequences and the coding sequence for a C-terminal triple HA-peptide tag.

Having considered several alternative options [29], we settled with a plan to undertake a full cut of prion gene alleles without repair template to harness the host-encoded NHEJ DNA repair system (**Fig 1A**). To maximize the potency of the all-in-one rAAV vector, it had to not exceed the packaging limit of ~4700 base pairs. This restricted its design to smaller Cas enzymes, which had to have high efficiency, minimal off-target cleavage, and a short PAM sequence. A promising candidate represented a Type II Cas9 endonuclease from *Staphylococcus lugdunensis (*Slu*)*. SluCas9 is approximately 25% smaller than the canonical *Streptomyces pyogenes* (Spy) Cas9 and had been engineered with four amino acid substitutions that increased its specificity, hence its high fidelity (HF) designation [33]. Moreover, SluCas9-HF was reported to exhibit a similarly broad target range as SpyCas9 based on its 5’-‘NNGG’-3’ protospacer adjacent motif, which provided greater flexibility in the selection of gRNA binding sites (**Fig 1B**).

To facilitate the nuclear targeting, detection, and expression of the endonuclease, its sequence was flanked by nuclear localization signals and followed by segments coding for three copies of a nine amino acid hemagglutinin (HA) tag and a polyadenylation signal borrowed from the bovine growth hormone (bGH) [33]. The cassette coding for the gRNA was inserted 3’ of and with the same directionality as the expression cassette for the endonuclease. For the assembly of rAAV vectors, we chose to co-transfect the vector alongside a packaging and helper plasmid into HEK293 cells (**Fig 1C**). The assembled vector was 4.7 kilobases in size and as such compatible with the administration of the gene therapy as an all-in-one rAAV vector (**Fig 1D**).

### Selection of prion gene-specific gRNAs

In selecting gRNA target sites for functionally ablating mouse or human prion genes, we focused on the first 150 nucleotides of the respective ORFs, i.e., Exon 2 in humans and Exon 3 in mice. The *in silico* selection of gRNA sequences was based on CRISPick [40], and ChopChop [41] tools. We also evaluated side-by-side a gRNA that was recommended by an early gRNA prediction algorithm, known as CRISPR Design [42]. The latter had previously been validated to generate an effective functional knockout of the mouse prion gene [43], whereas the gRNAs recommended by ChopChop (CC) and CRISPick (CP) had not yet been validated *in vitro*. All three gRNAs were estimated to have high on-target specificity and did not bind regions in the ORFs harboring common polymorphisms.

**Fig 2:**
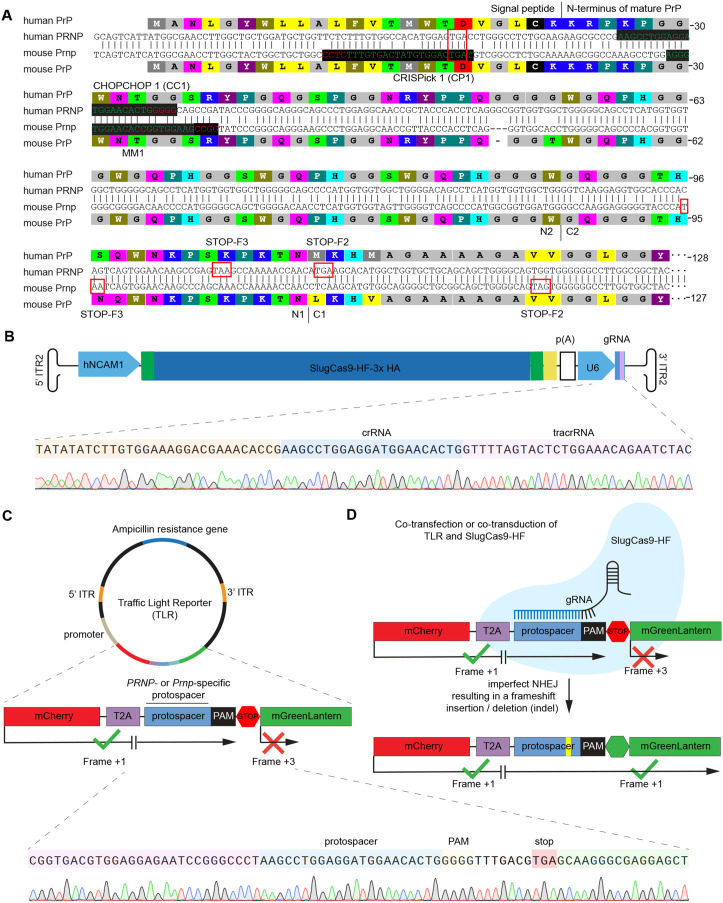
Selection of prion gene-specific gRNA. **(A)** Sequence alignment of the 5’ regions of ORFs of human and mouse prion genes, depicting top-ranked gRNA protospacers from CRISPick (CP1), ChopChop (CC1), and CrisprDesign (MM1). Termination codons within prion gene reading frames 2 and 3 (F2 and F3) are shown in red boxes. Note the proximity of these termination codons to the approximate position of natural endoproteolytic cleavage sites of α- and β-secretases, depicted with boundaries N2/C2 and N1/C1, respectively. **(B)** The Sanger nucleotide sequence read shown at the bottom of this panel validates the correct insertion of the top-ranked ChopChop1 protospacer sequence (CC1) targeting human PRNP into the guide RNA cassette (see Panel A for reference). **(C)** Depiction of elements of the prion gene-specific TLR, including its expression cassettes for red (mCherry) and green (mGreenLantern) fluorescent proteins, separated by a T2A peptide, prion gene-specific protospacer, PAM, and translation stop codon sequences. The sequence read at the bottom validates the correct insertion of CC1 into the TLR. **(D)** Experiment scheme following co-transfection or co-transduction of the therapeutic vector and the prion-gene specific TLR. **(D)** Cells that express the TLR will exhibit mCherry fluorescence but only the presence of gene editing events that lead to a shift into the + 1 reading frame in or near the protospacer sequence are expected to cause the co-expression of mGreenLantern.

Despite its relatively high error rate, the most common outcome of NHEJ-based repair of cut DNA sequences is the faithful restoration of the original DNA sequence. When this happens, the Cas9 can be redirected by the gRNA to the same sequence in a perpetual cycle of cut and repair until an erroneous insertion or deletion (indel) occurs that causes the gRNA to become a poor match for the then gene edited sequence. To understand the prion gene-specific consequences of such indels, it is instructive to map premature termination codons that may be shifted into the reading frame (**Fig 2A**). In both mouse and human prion gene sequences, frames 2 (F2) and 3 (F3) harbor nonsense codons that would terminate translation close to natural endoproteolytic sites, known to cause the release of N1 and N2 fragments [44].

When considering promoter choices, we sought to ensure that SluCas9-HF was particularly active in cells that express the prion gene. The use of the prion promoter was discouraged by its outsized length and prior reports which established that—unless a semi-synthetic construct is used [45]—it can be difficult to derive truncated versions of this promoter that retain its characteristic expression profile [46]. In contrast, the evolutionary older NCAM1 promoter has a more typical length and organization and had been shown to retain good expression when truncated to a 611 bp core promoter [47]. Based on the previously reported highly correlated expression of NCAM1 and PRNP genes in human tissues (FANTOM5 data set, RIKEN) [8,48], we selected the NCAM1 core promoter for this task to provide a gene editor whose expression aligns with the expression of PrP^C^ in brain cells (**Fig 2B**).

To optimize rAAV vectors for the intended application, it would be useful if their distribution and gene editing efficacy throughout the brain could be easily monitored. Fluorescence reporter systems have been used to address this kind of challenge before [49,50]. We decided to assemble a prion gene-specific “traffic light reporter” (TLR), which would light up the subset of brain cells that have been transduced and made to express their payload in red fluorescence based on the mCherry reporter (**Fig 2C**). To also monitor if the prion gene editing machinery is active in a given cell, we placed 3’ on the same reporter construct the coding sequence of the mGreenLantern reporter, which we separated from the mCherry coding sequence by a cassette coding for 1) a *Thosea asigna* virus 2A (T2A) self-cleaving peptide, 2) the stretch of the prion gene ORF that we were targeting with our protospacers, followed by 3) the respective PAM, and 4) a translation termination codon. With this design, the mCherry protein is produced as soon as the cell expresses the rAAV TLR vector, yet only after a successful gene editing event that shifts the termination codon out of frame and the mGreenLantern ORF into frame, will such cells also fluoresce green (**Fig 2D**).

### Purification of rAAVs using scalable AAVX-based affinity chromatography workflow

**Fig 3:**
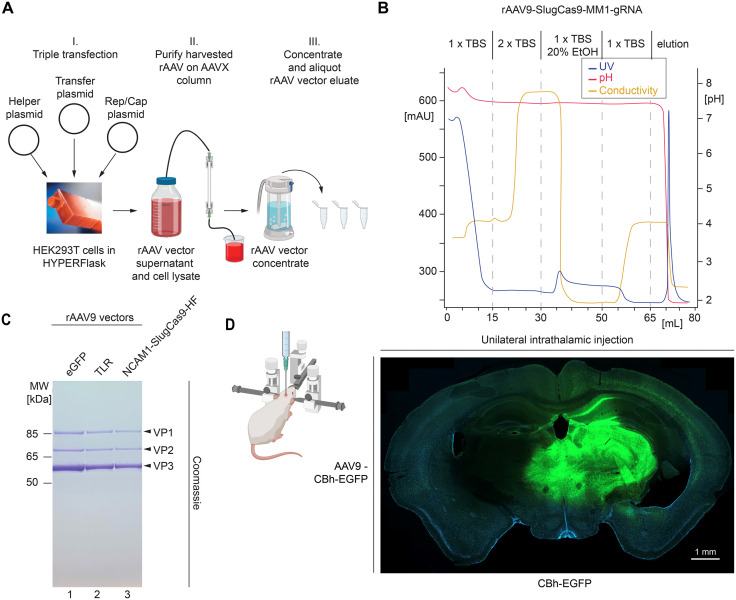
Purification of rAAV vectors using scalable AAVX-based affinity chromatography workflow and assessment of intracerebral delivery routes. **(A)** Cartoon depicting HYPERFlask and AAVX-based affinity chromatography method for the production and purification of high-titer rAAV9 vector preparations. **(B)** FPLC chromatogram of AAVX-based wash and elution of captured rAAV9 vectors, depicting UV, pH, and conductivity traces. **(C)** Coomassie-stained SDS-PAGE depicting purity of rAAV9 vectors. **(D)** rAAV vector coding for CBh-EGFP was packaged into AAV9 capsid, then administered by stereotaxic unilateral intrathalamic injection to C57Bl/6 mice. 21 days later, brains were coronally cut with a cryostat, then analyzed by fluorescence microscopy. Images are from sections cut −2 mm posterior to bregma and were co-stained with DAPI. The scale bar indicates 1 mm.

Given the widespread expression of the prion gene throughout the brain, the potency of any PrP^C^ lowering gene therapy will greatly hinge on the ability to transduce a large percentage of brain cells. The purity, integrity, and titer of rAAV vector preparations are major determinants that influence this desired outcome. Initially, we batch-purified rAAV vectors using conventional iodixanol or cesium chloride gradient-based preparation methods. With these approaches, batch-to-batch consistency and scalability were a concern, causing us to switch to a recently described rAAV vector affinity-capture method based on fast protein liquid chromatography (FPLC)-controlled AAVX matrix chromatography [37] (**Fig 3A**). With this method, the assembly of rAAV vectors was undertaken in HEK293T cells in HYPERFlasks. Filtered rAAV vector supernatants and lysates were captured and washed on AAVX resins coated with anti-AAV antibody fragments. Finally, the purified rAAV vectors were eluted by pH drop (**Fig 3B**), concentrated in stirred cell ultrafiltration units in the presence of low quantities of Pluronic F68 detergent for enhanced recovery. At this scale, the method consistently yielded titers exceeding 10^14^ viral genomes per batch that were free of contaminants when assessed by SDS-PAGE separation and Coomassie staining (**Fig 3C**).

For the administration of rAAV vectors in mice, we initially considered intrathalamic (IT) versus intracerebroventricular (ICV) routes. Others had shown that stereotaxic injections into the thalamus can facilitate the intracellular cell-to-cell distribution of rAAV vectors through cellular projections emanating from thalamic nuclei. In contrast, ICV injections were reported to facilitate widespread distribution through the network of extracellular cerebrospinal fluid cavities. When we injected rAAV vectors coding for the enhanced green fluorescent protein (EGFP) unilaterally into the thalamus we observed a pronounced expression of EGFP in the brain stem and hippocampal region, with lesser intensity of expression in outer cortical regions (**Fig 3D**). In contrast, ICV injections led to lower overall expression levels but more equal distribution throughout the ipsilateral hemisphere. In both instances, only low levels of green fluorescence were observed within the contralateral hemispheres that were not injected. Taken together, these experiments suggested that bilateral intrathalamic injections may provide potent expression of rAAV vector payloads in the deeper brain areas that are most early affected in prion diseases.

### Validation of rAAV vector coding for prion gene-specific traffic light reporter (TLR)

**Fig 4:**
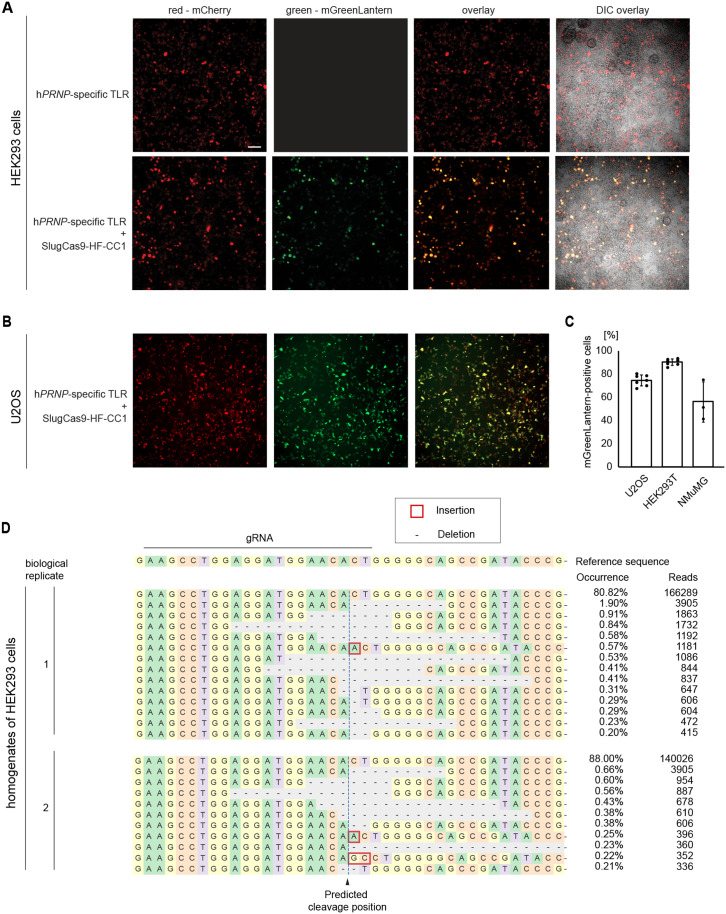
Validation of a fluorescent prion gene-specific traffic light (TLR). **(A)** Validation of the human *PRNP*-directed SlugCas9-HF-CC1 gene editing plasmid following its co-transfection with the *PRNP*-specific TLR plasmid into HEK293 cells, with cells transfected with only the TLR plasmid serving as negative controls. Scale bar represents 100 µm. **(B)** Evidence that the prion gene editing system tested in Panel A also works in other cell types, with human U2OS cells serving as an example. **(C)** Graph quantifying the relative proportion of transfected cells, recognizable by mCherry fluorescence, which also exhibit mGreenLantern fluorescence, and therefore appear yellow in the overlay. Error bars represent standard deviations and dots represent one biological replicate. **(D)** Complex *PRNP* gene edits in HEK293 cells transfected with NCAM1-CC1-SlugCas9-HF. Next-generation sequencing (NGS) results following paired-end amplicon sequencing of 150 bp *Prnp* amplicons. HEK293 cells were transfected with the SlugCas9-HF-CC1 plasmid. Three days after transduction, the cells were harvested, followed by genomic DNA extraction, *PRNP* gene edits were revealed by NGS amplicon sequencing, then tallied and characterized using CRISPResso software.

We initially tested the design of the TLR carrying the human *PRNP*-specific CC1 target sequence (TLR-CC1). To this end, we transfected HEK293 cells with the TLR-CC1 transfer plasmid alone or with both the TLR-CC1 and SluCas9-HF-CC1 transfer plasmids. Whereas fluorescence microscopy images obtained from HEK293 cells that were transfected with the TLR-CC1 plasmid alone exhibited red fluorescence only, the concomitant transfection of both plasmids led to the anticipated red and green fluorescence, which appeared as yellow in overlay images. The co-incidence of this red and green fluorescence appeared most apparent in cells that showed the brightest red fluorescence, perhaps reflecting that these cells were relatively more susceptible to transfection in general, and as such may have exhibited a higher propensity to take up both plasmids (**Fig 4A**). Similar results were also obtained when we transfected the same transfer plasmids to human osteosarcoma (U2OS) cells, thereby documenting that the host-encoded machinery required for gene-editing the TLR-embedded CC1 sequence was available in the two cell models tested (**Fig 4B**). The quantitation of signals corroborated this impression and established that ~75% of U2OS cells and ~90% of HEK293 cells, whose transduction led to mCherry expression were also expressing mGreenLantern. Next, we undertook analogous experiments with a transfer plasmid coding for the mouse *Prnp*-specific MM1 target sequence (TLR-MM1) and SluCas9-HF-MM1. This experiment led to 55% of cells exhibiting both red and green fluorescence (**Fig 4C**), indicating that the prion-specific TLR reporter can be deployed across ortholog boundaries so long as the embedded prion-specific sequence matches the prion sequence of the host. Although the number of gene-editing events per cell cannot be revealed by this approach, these data established that the prion gene-specific TLRs worked as intended and can be used for benchmarking improvements to the pre-clinical optimization of this prion gene ablation therapy.

To characterize in detail the prion gene edits introduced into HEK293 cells by the SluCas9-HF-CC1 editors, we undertook next generation sequencing (NGS) of prion gene amplicons comprising approximately ~150 nucleotides surrounding the target sites of the respective gRNAs. The NGS analysis of amplicons revealed that up to 19% of *PRNP* alleles were gene edited near the predicted CC1 target site. In this paradigm, gene edits observed were quite varied and included deletions and insertions, with the highest gene editing tallies observed for deletions spanning ≥ 8 nucleotides (**Fig 4D**).

### Proof-of-concept *in vivo* gene editing of the prion gene in mice

**Fig 5:**
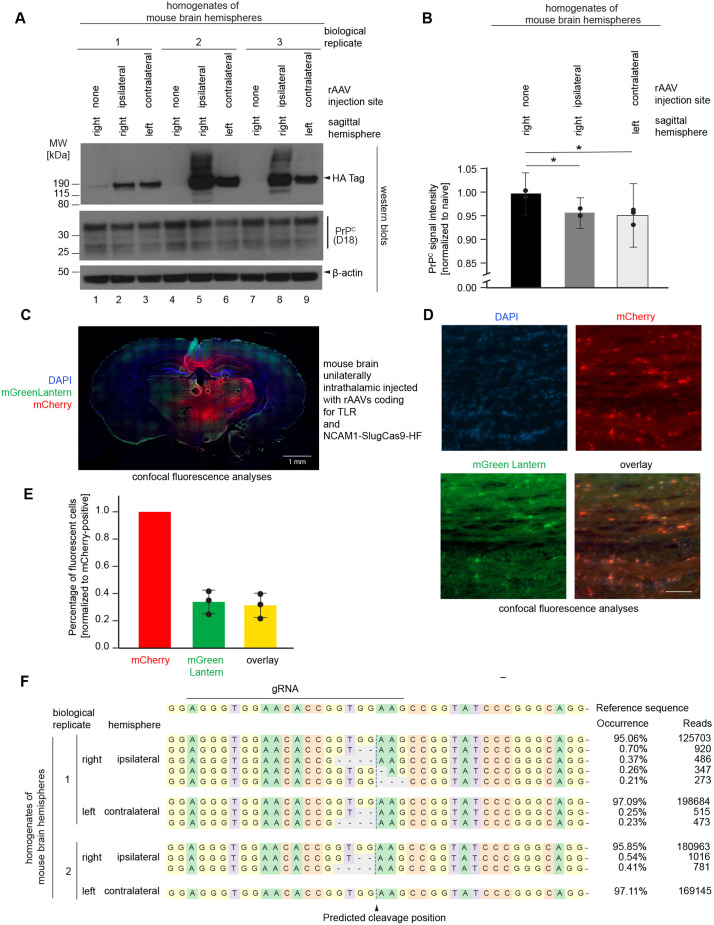
Proof-of-concept *in vivo* gene editing of the prion gene in mice. **(A)** SlugCas9-HF-MM1 packaged in the natural AAV9 capsid was administered to C57BL/6 mice by unilateral intrathalamic injection using a stereotaxic rig, with PBS-injected mice serving as a negative control. After 21 days, the mice were euthanized, subjected to transcardiac perfusion with PBS, and brains were cut sagittally into halves, then separately homogenized, and analyzed by western blotting. PrP^C^ was quantified using the antibody D18, SlugCas9-HF was quantified with an antibody directed to the 3xHA-tag it carried as a C-terminal fusion, and β-actin served as a loading control. **(B)** Quantitation of PrP^C^ signals from western blot in Panel A, corrected for equal loading using β-actin signal intensities and normalized to the signal from a naïve brain hemisphere that was not transduced. **(C)** Fluorescent-based analysis of AAV9 spread from ipsilateral intrathalamic injection site to contralateral brain half indicates predominant expression of the transduced transgene in ipsilateral hemisphere. The AAV9 vector coding for TLR, or a 1:1 mix of AAV9 vectors coding for TLR and SlugCas9-HF-MM1 were administered to C57BL/6 mice by unilateral intrathalamic injection. Brains were sectioned coronally and counter-stained with DAPI. The image depicts an overlay of mCherry, mGreenLantern and DAPI stains, documenting the limited distribution of the AAV9 vectors to the contralateral brain hemisphere. **(D)** Magnified fluorescence images of coronal section of C57BL6 mouse that was doubly transduced with TLR and NCAM1-MM1-SlugCas9-HF expressing rAAV vectors. Images are taken from the medial hippocampus. Scale bar represents 100 µm. **(E)** Graph depicting the proportion of red (mCherry) fluorescing cells that were also fluorescing green (mGreenLantern), indicative of the TLR reporter construct having undergone the intended SlugCas9-dependent NHEJ-mediated frameshift. Error bars represent one standard deviation. **(F)** Next-generation sequencing (NGS) results following paired-end amplicon sequencing of 150 bp *Prnp* amplicons. The SlugCas9-HF-MM1 expressing rAAV9 vectors were administered to mice by unilateral intrathalamic stereotaxic injection into the right hemisphere. Three weeks after transduction, the mice were sacrificed, and their brains were dissected into ipsi- and contra-lateral sagittal hemispheres. Following genomic DNA extraction of left and right hemispheres, *Prnp* gene edits were revealed by NGS amplicon sequencing, then tallied and characterized using CRISPResso software.

To evaluate a first implementation of the gene therapy in an *in vivo* model, we administered to three wild type (C57BL/6) mice the therapeutic rAAV vector that codes for SluCas9-HF-MM1. The objective of this pilot work was not to achieve the most robust functional knockout of the endogenous *Prnp* gene but to benchmark the performance of the rAAV vectors. To this end, 2x 10^11^ virus particles were unilaterally injected with a stereotaxic rig into the right thalamus using the coordinates we had previously established to achieve good rAAV vector spread. After a three-week incubation period, the mice were then deeply anaesthetized and subjected to transcardiac perfusion with PBS. Brains harvested were separated into ipsi- and contra-lateral sagittal halves, proteins extracted and analyzed by western blotting. Brain samples from naive age-matched wild-type mice served as negative controls. The level of expression of SluCas9-HF protein was inferred from the western blot signals detected with an anti-HA antibody. A close look at these signals revealed the expected expression bias, i.e., the ipsilateral brain tissue generated stronger signals than the contralateral brain tissue (**Fig 5A**). The negative control brain exhibited no signal or, in one instance, a spurious signal (through a cross-contamination during sample loading). Relative to brain extracts from naïve wild-type mice, the quantitation of total steady-state PrP^C^ signals revealed them to be slightly (5%), albeit significantly, reduced in all brain extracts from mice that had been administered the therapeutic gene therapy (**Fig 5B**). Counterintuitively, the levels of reduction in samples derived from either hemisphere were comparable in this small sample set, despite the higher level of SluCas9-HF expression in the ipsilateral hemisphere.

Next, to gain a better appreciation of the spatial efficacy of the gene therapy, C57BL/6 mice were stereotaxically co-administered the rAAV vectors coding for the therapeutic SluCas9-HF-MM1 construct and the *Prnp*-specific TLR-MM1 into their right thalamus. Three weeks later, the mice were fixed by transcardiac perfusion with 4% formaldehyde in PBS, rapidly dissected and postfixed in the same fixative for a limited time (one hour) to preserve fluorescence signals, then cryo-sectioned. As negative controls served age- and sex-matched naïve mice as well as mice who were administered the rAAV vector coding for the TLR but not the therapeutic SlugCas9-HF expressing rAAV vector. The analysis of brain slices by confocal microscopy revealed only mild background fluorescence in brain sections derived from naive mice, and the expected pronounced imbalance of fluorescence signals within the right hemisphere, with the strongest fluorescence signals detected within and surrounding the thalamic region. Robust, predominantly unilateral detection of the mCherry reporter was also detected in the hippocampal region and the hypothalamus, but relatively little reporter fluorescence was detected in brain cortical regions, including the parietal lobe (**Fig 5C**). In mice that had been co-transduced with the therapeutic rAAV vector, gene-editing was evident by robust mGreenLantern fluorescent signals in a subset of brain cells that also lit up with the mCherry reporter. Although a background unspecific sheen of green fluorescent could be seen throughout the brain when the green channel gain setting was raised digitally, no instances of a demarked green fluorescent was observed in cells that did not also exhibit red fluorescence. To quantify the relative proportion of cells that exhibited double fluorescence, fields of view were selected that each comprised approximately 100 demarked red signals, interpreted to represent brain cells that expressed the TLR reporter (**Fig 5D**). The separate tally of demarked red and green signals within these fields revealed that approximately thirty percent of transduced cells had undergone the intended gene edit (**Fig 5E**).

Although the purpose of this pilot *in vivo* experiment was not to maximize the prion gene edits throughout the brain, we were interested in assessing the complexity of gene editing outcomes and benchmark at the same time the efficacy. The biological source material for these analyses were mice that had been administered SluCas9-HF-MM1 by unilateral intrathalamic injection. To distinguish between ipsi- and contra-lateral gene edits, the brains of the mice were sagittally cut and *Prnp* amplicons within the two brain hemispheres were analyzed separately. Consistent with the approximately 5% reduction in steady-state PrP^C^ levels that we had observed by comparing signal intensities in western blot analyses (**Fig 5A**, **5B**), this analysis revealed a *Prnp* gene editing rate of close to 5% in ipsilateral hemispheres versus 3% in contralateral hemispheres (**Fig 5F**). As expected, based on ample precedents for NHEJ-based gene edits reported in the literature, the most common gene edits represented small nucleotide deletions 5’ or 3’ of the predicted SluCas9-HF-MM1 cut site.

### Retro-orbital administration improves AAV distribution within the brain, and the 9P31 capsid exhibits a higher and more consistent neuronal tropism in C57Bl/6 mice than PHP.eB capsids

**Fig 6:**
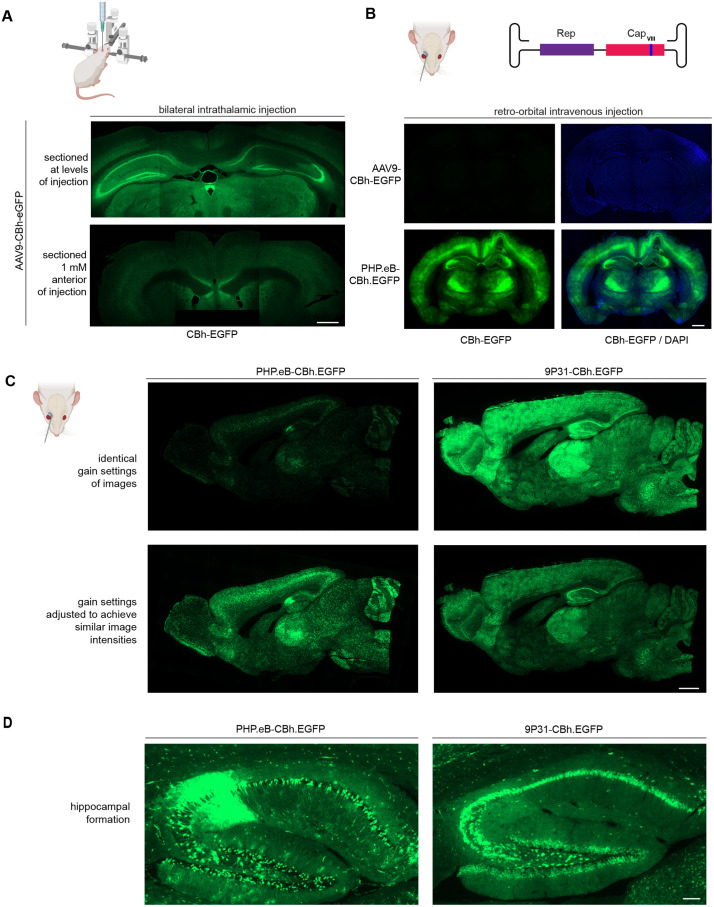
Retro-orbital administration improves AAV distribution within the brain, and the 9P31 capsid exhibits a neuronal tropism in C57Bl/6 mice. **(A)** 10^11^ AAV9 capsids (in 2.7 µL) coding for CBh-EGFP were administered to C57Bl/6 mice by bilateral stereotaxic intrathalamic injection at a rate of 50 nL/second at sites ±1 mm ML, −1.70 AP, −3.5 DV. The top panel shows a coronal section obtained immediately adjacent to the coordinates of the injection site. The bottom panel shows a section from the same brain obtained 1 mM posterior to the injection site. Sizing bar: 1 mm **(B)** 10^12^ AAV9 or PHP.eB capsids with a payload coding for CBh-EGFP were retro-orbitally administered to 6-week-old C57Bl/6 mice. Three weeks following the transduction, brains were perfused with 10% formalin, then coronally sectioned with a thickness of 16 µm (ML = 0.5) and mounted in DAPI-containing medium. **(C)** 10^12^ PHP.eB or 9P31 capsids coding for CBh-EGFP were retro-orbitally administered to 6-week-old C57Bl/6 mice. The subsequent processing mirrored brain shown in Panel B, except that the brains were sagittally sectioned with a thickness of 16 µm (ML = 0.5) and mounted in DAPI-containing medium. The top images were taken at identical gain settings and therefore depict differences in EGFP expression intensities. The bottom images show the same brain sections at intensity gain settings that equalize the overall fluorescent intensities to emphasize differences in the brain distribution of 9P31 and PHP.eB. Sizing bar: 1 mm **(D)** Magnified images of the hippocampal formations of brain sections shown at the bottom of Panel **C.** Note the scattered versus consistent EGFP fluorescent in mice that were transduced with PHP.eB versus 9p31 capsids. Sizing bar: 100 µm.

The pilot experiments thus far validated that the therapeutic rAAV vector is functional as intended but pointed toward limitations in its delivery. Whereas our data had shown that intrathalamic administered AAV9 vectors distribute throughout the thalamus and beyond in dorsoventral and mediolateral directions, we consistently observed limits in their spread to cortical areas and lacked data on how far anterior and posterior they spread. To address this, we next administered an AAV9-CBh-EGFP construct to the thalamus through bilateral intrathalamic injection, then studied cryosections which were cut coronally in immediate proximity to the injection sites or at distances of 1 mM anterior or posterior to the plain injection site. This experiment revealed that the spread from the thalamic injection site occurs poorly along the anterior-posterior axis (**Fig 6A**).

This study was initiated a few years ago. During its implementation, two pertinent developments occurred. 1) Retro-orbital injections were increasingly recognized as the most efficient and humane method for administering AAVs to mice intravenously. 2) Several recombinant AAV (rAAV) capsids became available that exhibit improved characteristics for crossing the blood brain barrier (BBB). To assess the gain in expression that can be achieved by replacing AAV9 with a capsid known to exhibit better brain penetrance, we initially worked with PHP.eB. To this end, we assembled PHP.eB-CBh-EGFP as described, then administered it retro-orbitally to C57Bl/6 mice, with AAV9 serving as a negative control. As anticipated [51], a profound gain in EGFP expression within the brain was the result, with AAV9-delivered EGFP expression almost undetectable in the brain(**Fig 6B**). We next tested several more recent capsid variants which had been shown to exhibit enhanced brain tropism in mice, including AAV.CAP-Mac [27], AAV.PAL2 [28], and 9P31 [52]. In our hands, both AAV.CAP-Mac and AAV.PAL2 led to minor enhancements in brain tropism relative to PHP.eB. However, consistent with prior data, when we administered 9P31-CBh-EGFP retro-orbitally to C57Bl/6, levels of EGFP expression exceeded the expression of this protein following retro-orbital administration of PHP.eB-CBh-EGFP approximately 7.5-fold [52,53]. Specifically, this value represents the difference in gain setting of the acquisition software (ZEN, Zeiss) that was required to achieve matching levels of EGFP green fluorescence throughout microscopy images (**Fig 6C**). Moreover, a close comparison of the tropism achieved with PHP.eB versus 9P31 capsids, revealed the latter to lead to a more consistent neuronal uptake, which can be documented in the hippocampal formation (**Fig 6D**).

### Improved but limited steady-state PrP^C^ levels in C57Bl/6 mice following transduction of 9P31-SlugCas9-HF-MM1

**Fig 7:**
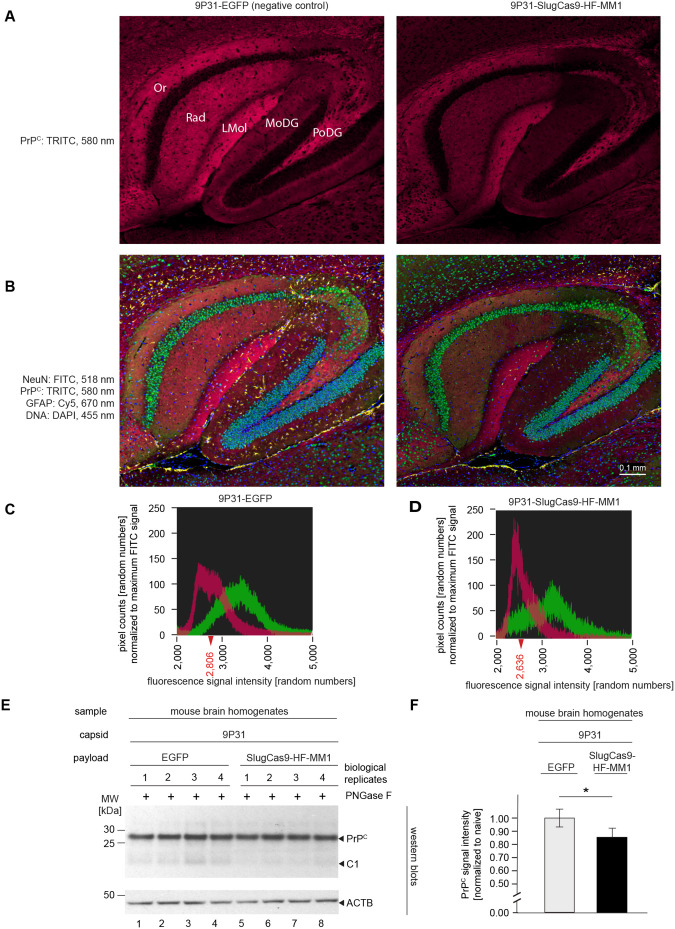
Retro-orbital administration of 9P31-SlugCas9-HF-MM1 in C57Bl/6 mice reduces steady-state PrP^C^ levels by 13%. **(A)** Hippocampal sections of C57Bl/6 mouse brains analyzed three weeks following retro-orbital administration of 10^12^ capsids of 9P31-EGFP (negative control) or 9P31-SlugCas9-HF-MM1. Digital gain settings were identical for all brain slices, including the representative data shown in this image. **(B)** Same images as above, showing additional fluorescence channels designated to the detection of NeuN, GFAP, and DNA. In this experiment all primary and all secondary fluorescent-labeled antibodies were premixed, and brain sections compared were exposed to the same premixes side-by-side. With this workflow, differences in PrP intensity can be revealed when digital gain settings are also fixed across samples. **(C and D)** Comparison of PrP (TRITC) and NeuN (FITC) signal intensities in mice transduced with 9P31-EGFP versus 9P31-SlugCas9-HF-MM1. The ordinate scale of the graphs are normalized to a value of 100 which depicts the maximum FITC signal pixel counts. Note that the TRITC signals are shifted to the left in this graph, i.e., lowered steady-state PrP levels in C57Bl/6 mice following 9P31-SlugCas9-HF-MM1 administration manifest in the detection of a larger proportions of pixels that exhibited lower signal intensity. **(E)** Western blot analysis of sagittal brain halves of treated brains, using beta-actin signals (ACTB) as a loading control. **(F)** Quantitation of steady-state PrP signal intensities (normalized to ACTB levels) in cells that were mock-treated with the 9P31-EGFP negative control vector or transduced with the 9P31-SlugCas9-HF-MM1 therapeutic construct. Note that the ordinate is interrupted in this graph to emphasize the significant differences (p < 0.05) in signal intensities.

Having validated the improved brain penetrance of 9P31 capsids, we next tested if the retro-orbital injection of 9P31-SlugCas9-HF-MM1 can be more efficient in reducing PrP^C^ levels than the approximate 5% PrP^C^ lowering achieved when we injected AAV9-SlugCas9-HF-MM1 intrathalamically (**Fig 5**). We also sought to capture if the PrP lowering manifested evenly in neurons targeted by 9P31 (**Fig 7A**). To this end, we implemented a multi-channel fluorescence detection workflow that can capture the expression profile of PrP^C^ alongside the levels of a neuronal marker (NeuN), an astrocytic marker (GFAP), and a nuclear DNA stain (DAPI) (**Fig 7B**). Through the pre-mixing of all primary antibodies used and the subsequent pre-mixing of secondary antibodies, the multi-channel analysis was to enable a comparison of PrP signals normalized against the signals of cell type-specific markers. When analyzed in this manner, the treatment with 9P31-SlugCas9-HF-MM1 caused PrP^C^ signals to shift to lower intensity in some but not all brain structures, as exemplified in sagittal images of the hippocampal formation (**Fig 7B**, **7C**). Within this formation, the lacunosum moleculare (LMol), polymorph dentate gyrus (PoDG), and oriens (Or) layer retained similar levels of PrP expression in 9P31-SlugCas9-HF-MM1 treated mice as the negative control mice, yet the radiatum (Rad) layer and molecular dentate gyrus (MoDG) were diminished in their PrP-dependent red fluorescence (compare left and right panels of **Fig 7A**).

Finally, when we undertook western blot analyses followed by quantitation of bands assigned to PrP^C^, we observed that steady-state levels of this protein were reduced by 13% in 9P31-SlugCas9-HF-MM1 treated mice when compared to mouse brains transduced with the negative control 9P31-EGFP vector (**Fig 7D**). A follow-on characterization of possible reasons for this still limited efficacy of our gene therapy revealed that our virus particle preparations had a higher empty to full ratio than we had estimated by qPCR. Specifically, a charge-detection mass spectrometry (CD-MS) analysis validated our 9P31-SlugCas9-HF-MM1 preparations to be relatively clean but also revealed that only 15% of these preparations represented full capsid. (S1–S2 Fig).

Taken together, these analyses established a first-generation of tools for functionally ablating the prion gene and a robust workflow for benchmarking the efficiency of this and future CRISPR-Cas9 based strategies for reducing PrP^C^ expression.

## Discussion

Here, we reported on the implementation of a CRISPR-Cas9-based gene editing platform for ablating the cellular prion protein in the brain. We described an all-in-one rAAV vector coding for the expression of a small Cas9 endonuclease and an empirically selected guide RNA targeting the prion gene. We also documented the design and use of a prion gene-specific traffic light reporter for monitoring gene editing efficacy. We described how these elements of our prion gene editing platform can be efficiently batch-assembled and purified to high titer preparations by adopting a rAAV vector affinity capture protocol. Evaluating different capsids, we found the 9P31 capsid to provide approximately 7.5-fold higher expression levels of a fluorescent marker protein than PHP.eB. Having selected a capsid that exhibits excellent brain penetrance, we moved from intrathalamic stereotaxic injections to retro-orbital administration because we observed this method to provide improved distribution. Finally, we characterized the gene editing efficacy of this pilot version of our platform and noted deficiencies in the fill ratios of our capsid preparations.

To our knowledge, this report is the first describing a therapeutic rAAV vector-based gene editor designed to ablate the prion gene that capitalizes on the error-prone host-encoded NHEJ program. We chose to rely for the expression of the Cas9 endonuclease on the NCAM1 promoter. Using a promoter whose activity correlates with the expression of the prion gene may be critical if the gene editing capacity of rAAV vector transduced brain cells is limited by the amount of SluCas9-HF that is expressed. In this scenario, it might ensure that the subset of cells whose NCAM1 promoter is most active (i.e., presumably the cells that also express the highest levels of PrP^C^) have the highest chance of being gene edited, leading to an outsized effect on total PrP^C^ levels. The ability to interfere with prion disease conversion and disease progression could be further augmented under such circumstances if the cells expressing the highest levels of PrP^C^ are also the most prone to undergoing the PrP^Sc^ conversion, a currently poorly understood but plausible scenario.

When we embarked on this study, the approach we selected was closest to the PrP^C^ lowering strategy based on rAAV vectors delivering prion gene-specific ZFRs that is being pursued by Sangamo [17,18], which blocks the initiation of prion gene transcription sterically through the binding of ZFPs to prion gene alleles.

While we assembled this report, a conceptually similar study was reported, which made use of a base editing strategy [22]. A downside of this strategy is that it relies on the delivery of a more complex gene editing platform coding for a catalytically inactive Cas enzyme fused to a base editor. Although such systems have been packaged as all-in-one rAAVs before, the larger size of base editing platforms relative to the mere expression of a catalytically active Cas9 can be expected to contribute to the overall immunogenicity of the gene therapy.

Both the base editor approach and the gene editing platform implemented here may cause nonsense-mediated decay (NMD) of transcripts. If the NMD process is not triggered (a plausible scenario because the PRNP open reading frame is not followed by additional exon-intron boundaries that can recruit exon junction complexes) [54], then the edited mRNA may give rise to the production of N-terminal PrP^C^ fragments. The best available evidence—based on a study of transgenic mice that overexpress the N1 fragment—suggests that such N-terminally truncated PrP^C^ fragments, solely composed of an intrinsically disordered segment of the protein, will be retained in the cytosol due to their inefficient translocation into the ER [55]. Should short N-terminal fragments escape this fate, get translocated into the ER, and escape the ER-resident quality control mechanisms, then one may expect them to be secreted because they would lack membrane embeddedness in the absence of a C-terminal GPI-attachment signal. In this scenario they may resemble the N1 fragments that are naturally secreted following endoproteolysis of cell surface-resident GPI-anchored PrP^C^ [56]. Although the full range of biological activities of such N-terminal PrP^C^ fragments is not known, their ability to compete with N-terminal binding domains present on cell surface PrP^C^, thereby sequestering certain ligands, is suggestive of beneficial and protective consequences in this scenario [57].

The development of any rAAV vector-based therapy faces challenges that relate to the tropism and immunogenicity of rAAV vectors. The ZFR-based strategy relies on the persistent availability of prion gene-specific ZFRs for sustained suppression of PrP^C^ expression but benefits from the relatively low immunogenicity of ZFRs due to their relatively small size and the broad evolutionary representation of transcription factors carrying these DNA binding domains in vertebrate genomes. Although all rAAV vector-based strategies that have been pursued to date can give rise to off-target effects that impact the transcription of genes other than *PRNP* itself, the use of a CRISPR-Cas9 gene editor poses larger risks of inadvertent immunogenic responses based on its relatively large size and recognition by the immune system as a foreign agent [21,58].

The few rAAV vector-based strategies for the treatment of any disease that have made their way into the clinic also suffer from high costs. For instance, the one-time treatment of spinal muscular atrophy [59] is made available at a cost of approximately US$ 2 million. Although economy of scale and competition are likely to bring down the costs of rAAV vectors in the future, high R&D costs, complex quality control, immune management, and risks associated with the administration of rAAV vectors, will continue to push up prices of these medicines, a hurdle that can be exacerbated when dealing with orphan diseases [60]. These thoughts are not new and are expected to become a major challenge for scientists and regulators and are merely recapitulated here as they would also apply to the roll-out of a future prion disease gene therapy whose pursuit we have embarked upon in this study.

This proof-of-concept study had limitations. The most obvious shortcoming of the prion gene ablation therapy presented is its limited efficacy. Despite these humbling beginnings, we are not discouraged as we attribute this outcome to technical challenges that can most likely be overcome with a second generation implementation of this treatment modality. For instance, we anticipate that an additional fractionation step that enriches the percentage of full capsid might go a long way to enhancing efficacy. Consideration should also be given to several other parameters that can have a strong influence on efficacy, including the gRNA selected for targeting the prion gene as well as the promoter and regulatory elements embedded within the capsid payload that can greatly improve the transport and processing of the SluCas9 mRNA. There are also reasons to be hopeful that re-administration of rAAV vectors may soon become possible if accompanied with refined immunosuppressive regimes or IgG depletion [61]. More experimental work will be needed to understand the specific fate that awaits gene products produced from *PRNP* genes mutated by indels and to characterize off-target effects of the gRNAs selected for this pilot study. Work by others have indicated that off-target effects can be minimized if care is taken to select the most target-specific gRNAs. Although we have employed *in silico* algorithms in the design of gRNAs tested to minimize off-target activity, these computational approaches are limited in their predictive power, and additional experimental work will be needed to establish that a gRNA short-listed for human use does indeed exhibit low propensity to direct off-target CRISPR-Cas9 editing. Because off-target effects are understood to vary greatly with the choice of Cas enzyme, specific gRNAs, and their genomic targets [40,62,63], we decided to postpone the necessary empirical off-target investigations until a time when a refined implementation of this combinatorial technology with the potential for human translation has been identified.

At that time, it will not only be critical to undertake efficacy study in prion-infected mice, but it will also be interesting to reassess consequences of the functional ablation of the prion gene. To date, much of what is known about prion gene deficiency phenotypes stems from observations of animals that never expressed the prion protein due to germline genomic changes that were either experimentally or accidentally introduced, a scenario that can favor mitigating compensatory biology. It is encouraging that recent gene therapy approaches, which targeted the prion gene, have not raised red flags in this regard [18] but this conclusion warrants further assessment in the form of refined cognitive and behavioral testing. Considering PrP’s known contribution to the morphogenetic programming of cells [64,65], as well as the activation of glia in prion diseases and in response to transduction with rAAV vectors, subsequent studies will want to capture maladaptive cellular reprogramming and neuroimmune dynamics [22,66], as well as crosstalk between these two types of biology.

The notion to explore if deployment of a CRISPR-Cas9 gene editor can provide sufficient levels of functional ablation of a gene-of-interest *in vivo* received independent validation in recent time, when it was reported that the CRISPR-Cas9-based, NHEJ-dependent ablation of ATTR and KLKB1 genes in the liver reduced plasma levels of their gene products by more than 90% [34,35]. One may expect that erroneous repairs of cut sites by NHEJ would manifest in indels of various lengths that would eliminate the natural register of an open reading frame in approximately two out of three instances. Detailed characterizations of the gene edits that were achieved in human livers in the two recent studies have not yet been reported. However, next-generation amplicon sequencing data undertaken with genomic DNA from the livers of cynomolgus monkeys revealed that NHEJ-dependent gene editing of the transthyretin gene led in 98.9% to a specific insertion of a single adenine at the SpyCas9 cut site, establishing how such high levels of gene ablation are achievable [67]. We are discussing these precedents here as a hopeful note, which may foreshadow that similarly effective target sites for the NHEJ-dependent gene ablation can also be found for other genes, including the prion gene, if more systematic efforts are invested in the identification of a Cas9/ gRNA combination that can drive the most efficient functional prion gene ablation.

## Conclusions

To date, our work on this program has been limited to proof-of-concept experiments. We have not yet shown that the gene editing therapy we described leads to survival extension. Even if we were to demonstrate promising results in a widely used prion disease infection animal paradigm, and methods for the safe and brain wide administration of gene editing rAAV vectors in human brains were to become available, such results would not be predictive of how well this approach would work in individuals that present with neurological symptoms in the clinic. The translation of these technologies to the treatment of humans afflicted with prion disease will need to be preceded by advances in the effective delivery of rAAV vectors to address the increase in scale that the human brain poses. The development of strategies that target the immune system to enable repeat rAAV vector administration may offer an alternative solution. The latter will certainly be needed before the approximately 2/3rd of individuals who are AAV seropositive due to prior exposure to natural AAVs can be considered for future rAAV vector-delivered gene therapy treatments.

## Supporting information

S1 Fig Charge detection mass spectrometry (CD-MS) establishes a fill level of approximately 15.1% for 9P31-SlugCas9-HF-MM1. (A) Mass spectrum of the 9P31-SlugCas9-HF-MM1 preparation used in this study generated on a CD-MS instrument. The spectrum validates the high purity of the capsids but also indicates that 73.5% of capsids were obtained empty, 11.4% were partially filled, and 15.1% of capsids were full. (B) Graph depicting the intensity distribution as a function of charge state.
(TIF)

S2 Fig Original and uncropped gel and western blot images shown in this manuscript. (TIF)
