# Supplementary figures and images for: Toward an all-in-one recombinant adeno-associated virus vector for functionally ablating the prion gene using CRISPR-Cas technology
Source: PLoS One. 2025 Nov 7;20(11):e0336578. doi: 10.1371/journal.pone.0336578 (PMC12594398; doi:10.1371/journal.pone.0336578)

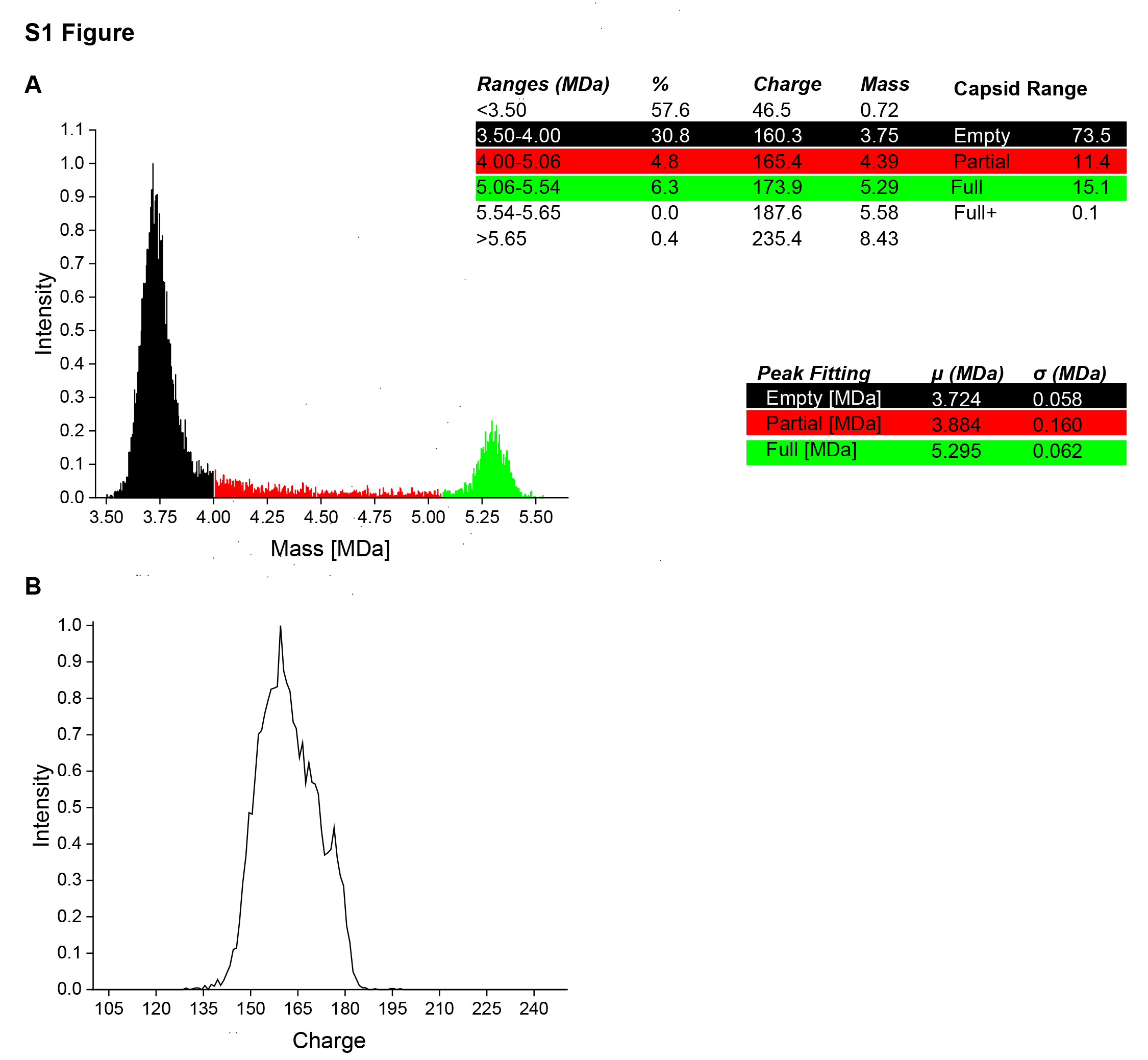

Supplement: S1 Fig — (A) Mass spectrum of the 9P31-SlugCas9-HF-MM1 preparation used in this study generated on a CD-MS instrument. The spectrum validates the high purity of the capsids but also indicates that 73.5% of capsids were obtained empty, 11.4% were partially filled, and 15.1% of capsids were full. (B) Graph depicting the intensity distribution as a function of charge state. (TIF) [file pone.0336578.s001.tif]

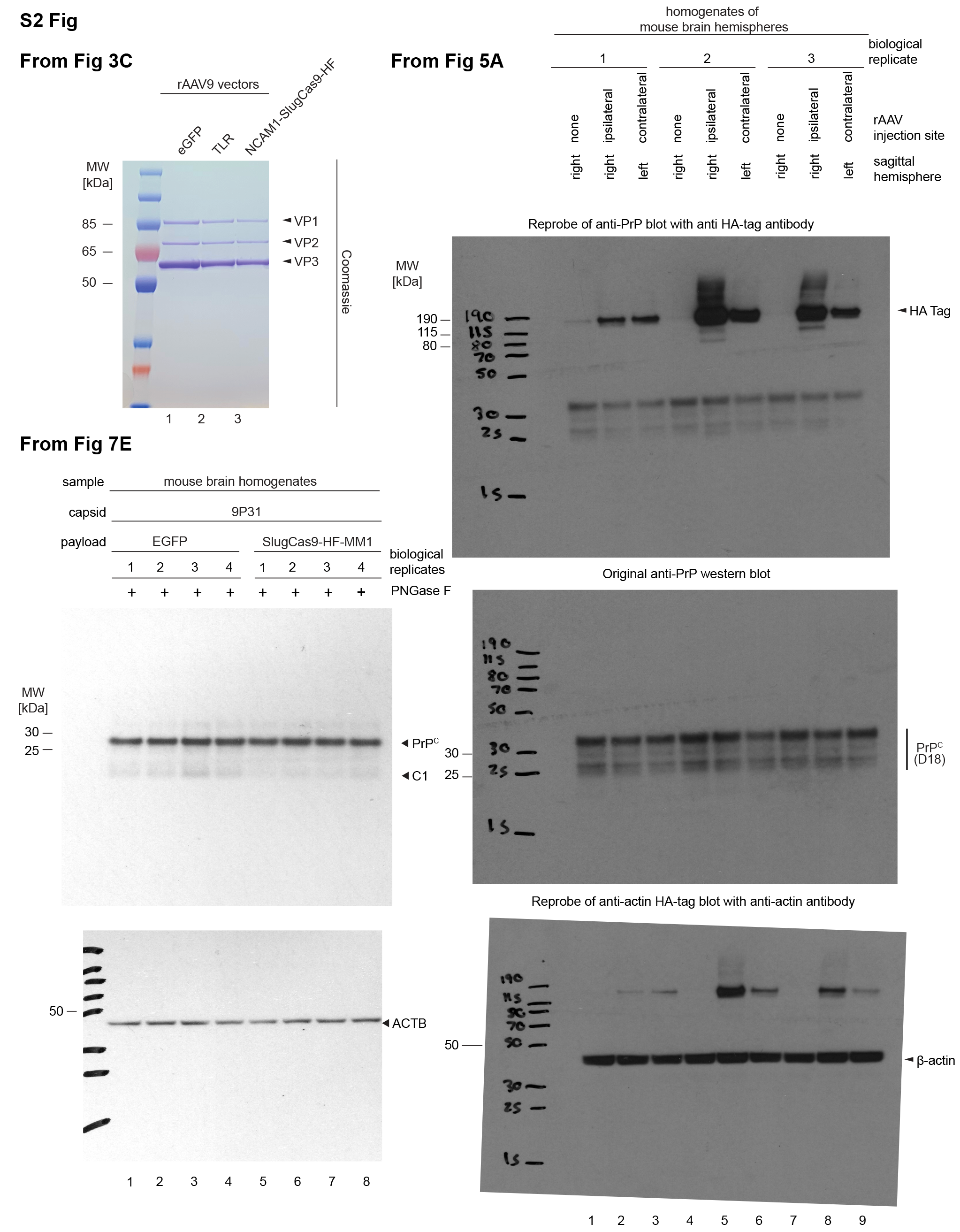

Supplement: S2 Fig — (TIF) [file pone.0336578.s002.tif]
